# Real‐world evidence to support regulatory decision‐making for medicines: Considerations for external control arms

**DOI:** 10.1002/pds.4975

**Published:** 2020-03-11

**Authors:** Mehmet Burcu, Nancy A. Dreyer, Jessica M. Franklin, Michael D. Blum, Cathy W. Critchlow, Eleanor M. Perfetto, Wei Zhou

**Affiliations:** ^1^ Center for Observational and Real‐World Evidence Merck & Co., Inc. West Point Pennsylvania, United States; ^2^ Real‐World Solutions IQVIA Cambridge Massachusetts; ^3^ Division of Pharmacoepidemiology and Pharmacoeconomics, Department of Medicine Brigham and Women's Hospital and Harvard Medical School Boston Massachusetts; ^4^ Center for Drug Evaluation and Research, Office of Surveillance and Epidemiology U.S. Food and Drug Administration White Oak Maryland; ^5^ Center for Observational Research Amgen Inc. Thousand Oaks California; ^6^ National Health Council Washington District of Columbia, and University of Maryland Baltimore (UMB), Baltimore, Maryland

**Keywords:** external controls, historical controls, pharmacoepidemiology, real‐world data, real‐world evidence, regulatory decision‐making

## Abstract

Randomized clinical trials (RCTs) are the gold standard in producing clinical evidence of efficacy and safety of medical interventions. More recently, a new paradigm is emerging—specifically within the context of preauthorization regulatory decision‐making—for some novel uses of real‐world evidence (RWE) from a variety of real‐world data (RWD) sources to answer certain clinical questions. Traditionally reserved for rare diseases and other special circumstances, external controls (eg, historical controls) are recognized as a possible type of control arm for single‐arm trials. However, creating and analyzing an external control arm using RWD can be challenging since design and analytics may not fully control for all systematic differences (biases). Nonetheless, certain biases can be attenuated using appropriate design and analytical approaches. The main objective of this paper is to improve the scientific rigor in the generation of external control arms using RWD. Here we (a) discuss the rationale and regulatory circumstances appropriate for external control arms, (b) define different types of external control arms, and (c) describe study design elements and approaches to mitigate certain biases in external control arms. This manuscript received endorsement from the International Society for Pharmacoepidemiology (ISPE).

**Key Points**

- There are clinical and regulatory circumstances where randomization is impractical or infeasible or unethical to conduct.
- Usually reserved for rare diseases and other special circumstances, external controls (eg, historical controls) are recognized as a possible type of control arm for single‐arm trials.
- In the absence of randomization, supporting regulatory decisions with external controls requires careful, detailed, and transparent planning and adherence to pharmacoepidemiological principles to minimize bias and confounding, and produce credible, actionable, and reproducible evidence.
- The paper discusses the rationale and regulatory circumstances appropriate for external control arms and defines different types of external control arms.
- The paper discusses specific recommendations to gauge the adequacy of the design elements of RWE in regulatory use of external control arms.

## 1 INTRODUCTION

Over the past several decades, randomized clinical trials (RCTs) have been the gold standard in producing clinical evidence of efficacy of medical interventions prior to their marketing authorization.^1^ But RCTs are expensive, time‐consuming, and often conducted among relatively homogenous patient populations with restrictive inclusion and exclusion criteria, limiting their generalizability to broader patient populations, and do not always provide answers to all questions pertinent to a medical product. The exponential growth of computing technology and the widespread availability of electronic health data have permitted real‐world data (RWD) to play an ever‐increasing role in health care decision‐making by a variety of stakeholders.^2^, ^3^ Examples include postmarketing safety assessment and surveillance of medical products by regulatory bodies, and cost‐effective decision‐making of medical product coverage by payers and health technology assessment bodies.^4^, ^5^, ^6^, ^7^ Postauthorization safety and effectiveness studies, based on pharmacoepidemiology methods and principles, are often considered as the foundation on the use of real‐world evidence (RWE) for postauthorization regulatory decision‐making by regulatory bodies around the globe. By definition, RWD are data relating to patient health status and/or the delivery of health care routinely collected from a variety of sources; and RWE is the clinical evidence about the usage and potential benefits or risks of a medical product derived from analysis of RWD.^8^

More recently, a new paradigm is emerging—specifically within the context of *preauthorization* regulatory decision‐making—for some novel uses of RWE from a variety of RWD sources to answer certain clinical questions.^9^ For example, enacted into law by the United States Congress in December 2016,^10^ the 21st Century Cures Act (P.L. 114‐255) (the “Cures Act”) requires the Food and Drug Administration (FDA) to develop a framework and guidance for evaluating RWE in drug regulation to support approvals of new indications for previously approved drugs, and to support or fulfill postapproval study requirements. Accordingly, in December 2018, FDA formally issued the framework for its RWE program,^8^ laying out the details of the multifaceted approach the agency is planning to undertake, which would include demonstration projects, stakeholder engagement, and guidance documents on specific topics to assist industry to develop RWE to support FDA regulatory decisions. Similarly, within the context of regulatory decision‐making by other regulatory agencies such as European Medicines Agency (EMA) and Health Canada, the concept of utilizing of RWD to support safety signal evaluation and risk management is not new, but there is an ever‐increasing interest in the use of RWE to support regulatory decisions across the product life cycle including development/preauthorization stage.^11^, ^12^ There are a growing number of European Union (EU)‐funded initiatives linked to RWE.^13^ In addition, published in 2015, EU Medicines Agencies Network Strategy to 2020 identifies RWE as a key player in supporting regulatory decisions for safe and effective use of medicines and bringing innovation to patients with unmet medical needs.^14^ In May 2019, China Center for Drug Evaluation also published draft guidance on key considerations in using RWE to support drug development.^15^

In approval of new molecular entities or label expansion, substantial evidence of efficacy of medical products is required from adequate and well‐controlled studies by regulatory bodies. Usually reserved for certain special circumstances, external controls (eg, historical controls) derived from RWD is also recognized by some regulatory bodies as a possible type of control arm for single arm trials to satisfy the substantial evidence standard for product approval.^8^ Similar to a randomized control arm in an RCT, external control arms represent a cohort of patients established to serve as controls to an intervention arm from a clinical trial. However, unlike in an RCT, these control patients are not randomized and are selected from data sources external to the single‐arm trial. In rare diseases or disease areas with high unmet need wherein randomization could be unethical or infeasible, external control arms have a growing role in supporting regulatory decisions on data generated from single‐arm nonrandomized trials.^16^, ^17^

In the absence of randomization, generation of an external control arm using RWD can be challenging and is subject to certain limitations, as it is difficult to fully control for confounding. Nonetheless, many biases can be attenuated using appropriate design and analytical approaches.^18^ The main objective of this paper is to improve the scientific rigor in the generation of external control arms derived from RWD. Here we (a) discuss rationale and circumstances appropriate for external control arms to support regulatory decision‐making, (b) define pros and cons of different types of external controls arms, and (c) discuss pharmacoepidemiologic design elements and approaches to mitigate bias that can be applied in the generation and analysis of external control arms. This manuscript received endorsement from the International Society for Pharmacoepidemiology (ISPE).

## 2 EXTERNAL CONTROL ARMS

### 2.1 Rationale and regulatory circumstances

In the assessment of efficacy and safety of an experimental medical product, the presence of a comparator (control) group is critical to understanding what happens to patients with similar characteristics and who would be subject to same conditions and procedures as in the experimental treatment group, but who do not receive the experimental treatment.^19^ In RCTs, randomization is conducted to allocate treatments to trial participants based on the presumption that all measured (observed risk factors) and unmeasured (unobserved risk factors) confounders would be equally distributed among the treatment arms in a study, satisfying the independence assumption of treatment assignment and ensuring that each participant has the same probability of receiving the experimental treatment or active control (or placebo).^19^

There are some clinical circumstances where randomization is impossible to undertake—due to ethical concerns and a state of clinical equipoise may not exist. Evaluated during planning of a randomized study, clinical equipoise is the state of uncertainty about not knowing which treatment or intervention would work better for study participants.^20^ These may include clinical settings with no available standard of care treatment or in life‐threatening disease areas with limited treatment options. There may be circumstances where randomization is impractical or infeasible. For example, in disease settings with high unmet need, a control arm may be perceived as a suboptimal treatment option by patients leading to challenges in the conduct of randomization—due to unwillingness of patients to enroll in or continue an RCT or due to the anticipated crossover of patients from the control arm to the experimental treatment arm during the course of a study. In other clinical settings, regardless of ethical concerns of randomization, randomization may not be feasible simply due to scarcity of patients (eg, rare diseases, or patient subgroups of a relatively common disease defined by a rare biomarker or mutation). For example, RWE from patient medical chart review in both the United States and Europe, serving as contemporaneous “benchmark” data, has supported the accelerated approval of Avelumab in Merkel cell carcinoma (a rare subtype of skin cancer) by FDA in 2017 based on data from a single arm clinical trial.^9^ In another example, RWE from a transplant registry was used to provide comparison data for similar patients enrolled in a single arm trial of Zalmoxis—a cell‐based treatment for a rare disorder—leading to its conditional authorization from the EMA.^9^

In the presence of ethical concerns or feasibility issues associated with randomization, RWD can serve as a source for external controls for efficacy and/or safety endpoints to help interpret data from a single arm trial, support expedited approval, or help label expansion of an approved therapy to additional disease states or subtypes defined by biomarkers, or by other patient and clinical characteristics. There are also other opportunities for external control arms to support regulatory decision‐making—even in RCTs. For example, external controls can help augment randomized control arms in RCTs by allowing smaller numbers of patients to be assigned (randomized) to control arms.^21^ These so‐called “hybrid” control arms (ie, mixture of randomized controls and external controls) can also help increase the efficiency of drug‐development process and may potentially allow for more resources to be used in the assessment of evidentiary gaps that may not be often otherwise addressed in traditional RCTs, including long‐term outcomes, additional endpoints that are more relevant to patients and payers, or endpoints that are augmented by patient or caregiver provided information.

### 2.2 Types of external control arms

External control arms are also called “synthetic” control arms as they are not part of the original concurrent patient sample that would have been randomized into the experimental or the control treatment arms as in a traditional RCT. External controls can take many forms. For example, external control arms can be established using aggregated or pooled data from placebo/control arms in completed RCTs or using RWD and pharmacoepidemiological methods. Pooled data from historical RCTs can serve as external controls depending on the availability of selected “must have” data, similarity of patients, recency and relevancy of experimental treatments that were tested, availability and similarity of relevant endpoints (eg, operational definitions and assessments), and similarity of other important study procedures that were conducted in these historical trials. It is important to note that using control data from historical RCTs still results in a nonrandomized comparison but has the advantage of standardized data collection in a trial setting and patients who enroll in clinical trials may have more similar characteristics than those who do not.

Depending on the regulatory and clinical context, RWE generated from external controls can serve as real‐world benchmarks (rather than as “formal” comparators) or as real‐world comparators.^9^ Real‐world benchmark data are useful for contextualization and to characterize the natural history of a disease, including treatment patterns and outcomes, but are more suited to support regulatory decisions when the regulatory threshold for action in the face of uncertainty is lower (eg, severe unmet need, scarcity of available patients). Real‐world “formal” comparators, on the other hand, require a more stringent planning and application of RWE that closely mirrors the patient population, inclusion and exclusion criteria, design, and analytical features of the single‐arm trial.

Determining whether a pharmacoepidemiological study is fit for regulatory purpose requires several considerations.^22^ The choice of data source, study design, and analytics should be tailored to the intended regulatory use of an external control arm (eg, new approval of a molecular entity, label expansion, and label revision); and should be based on the clinical context of that regulatory question (eg, prevalence/incidence of the disease, clinical equipoise, expected treatment effect, standard of care options, unmet need, benefit/risk, and uncertainty threshold considerations).^23^, ^24^, ^25^ Considerations should include the assessment of data quality and relevancy to fit intended purpose, along with other methodological (design and analytical) approaches to minimize bias and produce actionable and credible evidence for the intended regulatory purpose.

### 2.3 Pharmacoepidemiological design considerations

In most pharmacoepidemiological research using existing RWD, treatment assignment cannot be randomized; thus, the receipt of treatment may be dependent on multiple factors—including patient sociodemographic and clinical characteristics, insurance status, prescriber preference, and geographic or institution related variations in the practice of medicine. In the absence of randomization, the design of an external control arm with RWE should be constructed in view of the intended regulatory use, regulatory requirements, clinical context, timeline for evidence generation, and availability of appropriate and sufficiently high‐quality data sources.

#### 2.3.1 Contemporaneous, historical, or hybrid cohorts

As summarized in Table 1, the options for an external control include the use of: (a) contemporaneous cohorts with prospective data collection (eg, registry approach to support single‐arm trials, or augmenting control arms in RCTs), (b) contemporaneous cohorts with retrospective data collection, (c) historical cohorts using retrospective data, and (d) other hybrid approaches that may include mixture of both historical and contemporaneous cohorts.

**Table 1:** Cohort and data collection options for an external control arm generated using real‐world data (RWD)

	External control cohort inception date	Possible types of data collection	
		Retrospective RWD collection	Prospective RWD collection
Contemporaneous external control	On or after the first patient enrollment in the clinical trial	✓	✓
Historical external control	Before the first patient enrollment in the clinical trial	✓	
Hybrid external control	Varies	✓	✓

Contemporaneous vs historical cohorts differ in terms of their timing for cohort inception. For example, if an external control arm is constructed using RWD to support a single‐arm clinical trial with a first patient enrollment in 2016, a historical control arm could be created using RWD collected before first patient enrollment in the clinical trial (ie, before 2016). In contrast, a contemporaneous control arm could be created if RWE was generated on or after the first patient was enrolled (eg, using RWD collected in 2016 and onward). To account for any potential temporal changes—including changes in the standard of care, medical practice or procedures, diagnostic criteria, and patients' beliefs and health behaviors—contemporaneous control cohorts are preferable to historical controls. A particularly relevant potential change in medical practice is a change in who is eligible for treatment at all, which may drastically change severity of disease of patients included. However, there may be circumstances where the generation of external cohorts with contemporaneous data is not feasible, including the lack of availability of recent high‐quality data, or scarcity of patients necessitating the use of historical data from multiple contiguous years. In these circumstances, the use of historical external controls may be acceptable under the condition that there were no large temporal shifts in the standard of care, medical practice, patient management, or patient characteristics that are noteworthy. When appropriate, sensitivity analyses may also be considered by using analytical methods that place more weight on more recent data points in a historical cohort to reduce time‐trend bias.

Contemporaneous cohorts can be established at the end of the single‐arm trial using recent retrospective data or at the start of the single‐arm trial with prospective designs where patients are followed‐up and evaluated in real‐time after the study conception and initiation. In the absence of required data elements in accessible real‐world retrospective data sources (eg, high‐quality endpoints, lack of details on diagnostic criteria), prospective “registry” type studies may be utilized to address a specific regulatory question—albeit often more expensive and time consuming. Similarly, certain study questions may necessitate the use of a hybrid approach by collecting retrospective RWD of interest and also simultaneously establishing a prospective external control arm to collect additional outcomes that may not be readily available in existing RWD sources (eg, patient provided information and perspectives,^26^, ^27^, ^28^ patient reported outcomes, clinical assessments confirming diagnosis and severity, and other established clinical endpoints). Likewise, in some settings, hybrid approaches may also be used to augment controls in RCTs with additional real‐world controls, enabling fewer patients to be recruited into the randomized control arm in an RCT (ie, enabling the conduct of smaller RCTs). Confounding can still be an issue in these hybrid‐randomized settings, and thus, the sociodemographic and clinical characteristics of these supplemental RWD controls should be assessed carefully in the design and execution stages.

#### 2.3.2 Benchmark vs formal comparator data

The choice of design elements of an external control arm should also be weighed in the context of available sources of data and patients. If the regulatory purpose of an external control arm is to provide benchmark or supplemental historical data from diverse populations treated in real‐world settings,^9^ the external controls may be defined more broadly than in the treated group in single‐arm trials. On the other hand, if the purpose is to establish a formal comparator group, the external controls ought to be closely similar to the treated patients in single‐arm trials.

Regardless of the choice of an external control arm, selection bias and channeling bias could arise if the external comparator patient cohort was selected exclusively from clinically different populations (eg, differences in disease severity, disease duration, prior treatments, other patient related characteristics, such as age, gender and, race/ethnicity) than the single‐arm trial populations.^29^ As usually the research question is to compare the outcomes between experimental arm and control arm, the external control arm may not need to represent the general patient population but instead should represent a similar population receiving the experimental treatment. To mitigate confounding by indication/disease severity, the external cohorts should closely mirror the inclusion and exclusion criteria, and treatment and medical care history of patients in the single‐arm trial, recognizing that there may be limited use in real‐world practice of some laboratory tests and clinical measures used in RCTs. In addition, while assessed during routine visits, some clinical parameters do not get routinely recorded in clinical notes and may not be available for research in RWD sources (eg, Eastern Cooperative Oncology Group (ECOG) performance status or other functioning scores, results of laboratory tests conducted elsewhere). It is important to consider that filtering patients based on strict inclusion and exclusion criteria can potentially result in small sample sizes. Finding a balance between applying a strict set of inclusion and exclusion criteria and confounding adjustments may be needed in a small sample situation.

These biases may continue to persist in special circumstances, and the application of identical limits may not always result in the identical distribution of patient characteristics. Even after applying the same inclusion and exclusion criteria, the distribution of patient characteristics from an external control arm may be quite different from that of a single‐arm trial. For example, mean age may be quite different, even if age restrictions are the same. It is important to note that on rare occasions, RWD sources may also comprise patients recruited into other clinical trials who may significantly differ in terms of disease severity than those not enrolled in clinical trials or who may have more comorbidities or are frail. Treatments received by patients enrolled in clinical trials may also not be available or recorded in RWD sources. Additionally, specifically in regulatory uses of RWE for label expansion of a medical product with a prior market authorization, RWD may also comprise patients with off‐label use of that medical product or other related products. The feasibility of excluding or including these patients into the contemporaneous or historical external cohorts, and the likelihood that they could exert substantial influence over the effect estimate, should be assessed carefully in consideration of any potential bias related to available treatment options in routine health care settings, including clinical trial treatment options. The potential bias by including off‐label users would be, however, limited unless this subgroup is large and has very different expected risk outcomes.

While the use of advanced analytical and statistical approaches in generating RWE using external control arms is not within the main focus of this paper, it is important to note that further advanced analytical options may also be considered to mitigate confounding.^25^, ^30^, ^31^ Examples include propensity score matching or inverse probability of treatment weights.^32^, ^33^ It is, however, likely that the number of covariates available for analytical adjustment may be limited by the number of covariates concurrently available both in the clinical (trial) data set and in RWD.

#### 2.3.3 Sources of RWD and target patient population

The choice of source(s) of RWD should be assessed based on several factors, including the availability of appropriate clinical endpoints, availability of relevant patient groups or subgroups, duration of available follow‐up in a given data source, extent of missing data for key “must‐have” data, and accuracy of linkage, if any, to other relevant data sources.^24^, ^34^ For example, many single‐arm trials are conducted internationally with patients being recruited simultaneously across multiple clinical research sites. In establishing an external control arm to an international single‐arm trial, if the available standard of care treatment options, diagnostic criteria, or patient characteristics are vastly different across regions or countries or care settings, the use of an established secondary data source from a single region or country may not be sufficient as a comparator for the treated arm overall but could add valuable country‐specific information. In certain cases, the use of geographically diverse international data, potentially from multiple sources (eg, global chart review studies), may be desirable. A main driver for evaluation of comparability is whether the health systems for each locale are likely to systematically capture the core data of interest. It should be noted that use of local comparator treatments (often referred to as “standard of care”) means that the treatments used in each geography or care site might differ substantially based on cost and availability. Additionally, if the endpoints in a single‐arm trial are defined in a clinically complex manner, structured data from electronic health records alone or administrative claims may not sufficiently capture the required granular data, and accordingly, an analysis of original sources of RWD, for example, through chart abstraction by trained clinical personnel or through review of unstructured electronic health record data, may be needed to establish high‐quality real‐world endpoints. Primary data collection may be preferred in instances when the endpoints of interest are not available in RWD sources, such as patient‐reported clinical outcomes and health‐related quality of life measures.

#### 2.3.4 Index date, follow‐up, and endpoints

Index date and follow‐up procedures represent other important concepts for consideration in establishing external controls. In clinical trials, the index date is usually defined as the date on which patients receive their first treatment administration after meeting certain inclusion and exclusion criteria. The time elapsed from diagnosis or from the start of recruitment process to the start of drug therapy is considered immortal, as patients must stay alive before the drug is administered. To mitigate any potential immortal time bias,^35^, ^36^ the algorithm for index date in an external control should be constructed, as feasible, to mimic the scenarios in the corresponding single‐arm clinical trial. The inclusion and exclusion criteria should not be based on any information ascertained after the start of follow‐up. While the delays to starting treatment in a clinical trial are generally short, immortal time bias can be problematic in external controls specifically in disease settings wherein mortality rates are high (eg, advanced/metastatic cancers) if index date definitions are not closely aligned. If an external control arm is selecting and following patients who are eligible for treatment but are not treated, immortal time bias may arise, as patients who would have survived to receive treatment would be systematically underrepresented in this external cohort. To mitigate bias, patients in external control should be selected from similar time periods in their disease course or history compared with patients in the trial.

Depending on the clinical context and endpoints to be assessed, the intensity and frequency of follow‐up visits in real‐world settings should also be evaluated carefully to ensure that data required for endpoints (eg, imaging data, laboratory test results) would be available and assessed in a similar fashion as in the single‐arm trial to mitigate performance bias. Endpoints used in clinical trials may not always be available or assessed in similar fashion in real‐world setting. For example, in oncology, clinical parameters or imaging data needed to assess objective response rate or progression free survival may not be always available in RWD sources. Therefore, to mitigate detection bias, developing and validating endpoints that can be used in real‐world studies are critical. In addition, certain biases related to differential censoring or loss‐to‐follow‐up (eg, attrition bias) as compared with the clinical trial population should be evaluated to implement necessary and appropriate analytical adjustments, for example, censoring weights.^37^

#### 2.3.5 Transparency, reproducibility, compliance, and ethics

A transparent and reproducible process for establishing external controls requires a detailed scientific protocol with clear objectives, description of study population (eg, inclusion/exclusion criteria, disease definition, and diagnostic criteria), description of data source and elements, study design, study duration, study endpoints, analytical plan, operational execution plan, planned subgroup and sensitivity analyses, anticipated limitations and challenges, and quality check procedures. Some recommendations and guidelines have been developed pertinent to methodological standards, data quality, relevancy, transparency, reproducibility, and stakeholder engagement.^22^, ^38^, ^39^, ^40^, ^41^, ^42^, ^43^ In addition to methodological and scientific standards, the process for establishing external controls will need to be in full compliance to all applicable local, national, and international law and regulations, the International Council for Harmonization of Technical Requirements for Pharmaceuticals for Human Use (ICH) guidelines and principles, and all applicable ethical, legal, and regulatory standards and operating procedures. In the case of analysis of “formal” comparator data, the ethics and compliance aspects of incorporating clinical trial data with RWD should be thoroughly examined (eg, informed consent, patient data protection). For example, incorporating individual patient‐level data from a single‐arm clinical trial data with external control RWD may be necessary to conduct direct statistical comparisons or conduct advanced statistical matching techniques, such as propensity score matching.

## 3 CONCLUSIONS

The potential benefits of establishing external controls using RWE to support regulatory decision‐making include providing evidence in circumstances when conducting traditional RCTs is unethical, impractical, or infeasible; supporting evidence development of marketed medical products for label expansions; increasing efficiencies of evidence development for regulatory purposes and expediting access of medical products to patients; and enabling supplemental evidence development that is more relevant to patients, providers, payers, and policy makers. The growing emphasis on the role of RWE in decision‐making by regulatory agencies has fueled a new optimism to achieve these goals and provided an impetus for a new regulatory framework.

The acceptance of external control arms by regulatory agencies to support specific regulatory decisions may differ across therapeutic areas and clinical contexts. Several disease areas can uniquely benefit from the use of RWD in establishing external controls, given the randomization‐based challenges associated with investigating rare diseases or diseases with high unmet need, particularly in view of the ever‐increasing number of patient subpopulations defined by specific genetic mutations or biomarkers.^44^, ^45^ In the absence of randomization, supporting regulatory decisions with external controls requires careful, detailed, and transparent planning and adherence to pharmacoepidemiological principles to minimize bias and confounding, and produce credible, actionable, and reproducible evidence. In light of the changing regulatory landscape, continued efforts by stakeholders are needed to harmonize principles for regulatory use of RWD for external control arms. We start by offering specific recommendations to gauge the adequacy of the design and analysis of RWE in regulatory use of external control arms.

## ETHICS STATEMENT

The authors state that no ethical approval was needed.
